# Daily Rapid Antigen Exit Testing to Tailor University COVID-19 Isolation Policy

**DOI:** 10.3201/eid2812.220969

**Published:** 2022-12

**Authors:** Rebecca Earnest, Christine Chen, Chrispin Chaguza, Anne M. Hahn, Nathan D. Grubaugh, Madeline S. Wilson, for the Yale COVID-19 Resulting and Isolation Team

**Affiliations:** Yale School of Public Health, New Haven, Connecticut, USA (R. Earnest, C. Chaguza, A.M. Hahn, N.D. Grubaugh);; Yale Health, New Haven (C. Chen, M.S. Wilson);; Yale University, New Haven (N.D. Grubaugh)

**Keywords:** COVID-19, SARS-CoV-2, coronavirus disease, severe acute respiratory syndrome coronavirus 2, respiratory infections, zoonoses, viruses, epidemiology, direct-to-consumer screening and testing, patient isolation, student health services, United States

## Abstract

We evaluated daily rapid antigen test (RAT) data from 323 COVID-19–positive university students in Connecticut, USA, during an Omicron-dominant period. Day 5 positivity was 47% for twice-weekly screeners and 26%–28% for less-frequent screeners, approximately halving each subsequent day. Testing negative >10 days before diagnosis (event time ratio (ETR) 0.85 [95% CI 0.75–0.96]) and prior infection >90 days (ETR 0.50 [95% CI 0.33–0.76]) were significantly associated with shorter RAT positivity duration. Symptoms before or at diagnosis (ETR 1.13 [95% CI 1.02–1.25]) and receipt of 3 vaccine doses (ETR 1.20 [95% CI 1.04–1.39]) were significantly associated with prolonged positivity. Exit RATs enabled 53%–74% of students to leave isolation early when they began isolation at the time of the first positive test, but 15%–22% remained positive beyond the recommended isolation period. Factors associated with RAT positivity duration should be further explored to determine relationships with infection duration.

In December 2021, the Centers for Disease Control and Prevention (CDC) reduced the recommended COVID-19 isolation period for the general population from 10 days to 5 days after symptom onset or a positive viral test (*1*). To end isolation, persons must have resolving symptoms and wear a mask for an additional 5 days; however, a negative exit test was not required. The rationale for the shortened isolation was based on practical and scientific considerations; namely, weighing the societal and economic burdens against the diminishing risk for transmission as a positive person proceeds through the infection. The CDC revised its guidelines as the SARS-CoV-2 Omicron variant rapidly grew to dominance in the United States, increasing from 1% to >50% of reported sequences over a 2-week period in December 2021 (*2*). Early analysis suggested different viral dynamics for Omicron versus Delta: lower peak viral RNA and shorter clearance periods for Omicron, but similar proliferation times and clearance rates (J.A. Hay et al., unpub. data, https://doi.org/10.1101/2022.01.13.22269257). Because the recommendations were based on estimates for earlier SARS-CoV-2 variants, more data were needed to understand their appropriateness for Omicron.

The updated guidance acknowledged the possibility of onward transmission after a 5-day isolation, citing an earlier UK modeling study estimating that 31% of persons remain infectious after day 5 (D. Bays et al., unpub. data, https://doi.org/10.1101/2021.12.23.21268326). Recent literature on exit testing from an Omicron-dominant period further indicates that high proportions of persons remain potentially infectious beyond day 5 (*3*; E. Landon et al., unpub. data,-https://doi.org/10.1101/2022.02.01.22269931; S.B. Nelson et al., unpub. data, https://doi.org/10.1101/2022.02.11.22270843). Studies of managed isolation programs through schools or employers found positivity of 31%–58% by rapid antigen test (RAT) on days 5–9, although daily testing among all persons was not conducted. Near-daily PCR testing found a day 5 positivity range of 39%–52% (J.A. Hay et al., unpub. data).

Although PCR tests are a preferred initial diagnostic option because of their high sensitivity, RATs are more suitable for exit testing when the goal is to determine when a person is likely no longer infectious. High PCR sensitivity may result in positive tests beyond the infectious period, leading to unnecessarily long isolations (*4*,*5*). RAT positivity is generally associated with culturable virus, which itself is often a proxy for infectiousness (*5*–*7*). In addition, to investigate concerns that RATs may have inferior performance for Omicron versus Delta infections, a study compared same-day positivity between the variants, finding similar sensitivity of RAT and PCR tests (*8*). Last, RATs have the advantage of relative affordability, fast turnaround time, and at-home self-administration compared with PCR tests, making them the only viable exit test option for much of the population (*9*,*10*).

In this study, we aimed to address the evidence gaps regarding changes in daily RAT positivity, factors influencing RAT positivity duration, and how exit RATs toward the end of isolation can be used to tailor isolation periods on the basis of risk. We evaluated daily RAT data from 323 persons who initially tested positive for SARS-CoV-2 during January 1–February 11, 2022, and were in a university-managed isolation program in Connecticut, USA. We designed our study to answer 2 questions: the percentage of SARS-CoV-2-positive persons that remained positive via RAT on day 5 of isolation and each subsequent day until testing negative; and the factors associated with RAT positivity duration. The Institutional Review Board (IRB) from Yale University Human Research Protection Program determined that the use of information, including information about biospecimens, is recorded by the investigator in such a manner that the identity of the human subjects cannot readily be ascertained directly or through identifiers linked to the subject and thus is exempt from IRB review of human subjects research (IRB protocol 2000032111).

## Methods

The university required undergraduate students to screen at arrival on campus and then twice weekly on designated days. SARS-CoV-2–positive students isolated and participated in mandatory daily rapid antigen self-testing beginning on day 5 after diagnosis until they tested negative. We defined diagnosis (day 0) as the earliest positive or inconclusive test date. All inconclusive persons subsequently tested positive. Excluding 27 persons whose results were by external PCR or home RATs, all received diagnoses by Clinical Research Sequencing Platform SARS-CoV-2 real-time reverse transcription PCR diagnostic assay (*11*). Trained staff observed the exit testing process and confirmed the result. Upon testing negative, students ended isolation but continued mandatory masking until day 10. All rapid antigen testing was conducted using the Quidel QuickVue At-home COVID-19 test (https://www.quidel.com), a lateral flow immunoassay that qualitatively detects the SARS-CoV-2 nucleocapsid protein antigen (*12*). The test received a US Food and Drug Administration–granted emergency use authorization for prescribed home use with patient-collected anterior nares swab specimens; it has a sensitivity of 84.8% (95% CI 71.8–92.4) and specificity of 99.1% (95% CI 95.2–99.8).

We used R version 4.0.5 and RStudio version 1.4.1106 for our analyses (*13*). We calculated the percent still positive as the number of positive persons each day divided by the total number of positive persons. To assess prognostic factors associated with the time to event (i.e., testing negative), we coded an accelerated failure time (AFT) lognormal regression model using the R package survival version 3.2–13 (*14*,*15*). We selected the AFT model for its suitability for interval-censored data (*16*). Because students entered the study on day 0 but were not rapid tested until day 5, any persons testing negative on day 5 were interval censored; their true negative time was between day 1 and day 5. We compared model fits using various distributions and selected the fit resulting in the lowest Akaike information criterion value. We exponentiated the regression coefficients to calculate the event time ratio (ETR), which is associated with prolonged RAT positivity duration when >1 and decreased duration when <1. An ETR of 1 signifies that RAT positivity duration does not differ by covariate level. We checked the assumption that the ratio of survival times (i.e., the ETR) is constant for all fixed probabilities of S(t), the survival function, using the R package AFTtools version 0.2.1 to inspect QQ plots generated for each covariate level comparison (*17*).

## Results

Our study population comprised primarily students 18–22 years of age living in university dormitory housing (N = 323) (Table 1). Among them, 63% self-reported symptoms before or at diagnosis. Symptomatic persons reported symptom onset a median of 0 days (IQR 0–1.25 days) before their initial test in the last negative test <4 days and last negative test 5–9 days groups and 1 day (IQR 0–4 days) before in the last negative test >10 days group. We did not track symptoms beyond diagnosis, although 18/205 symptomatic persons had a symptom onset date 1 day after diagnosis, potentially reflecting when they received their results and discussed symptoms. We found that 7% had a confirmed SARS-CoV-2 infection >90 days before their recent diagnosis: 62% of those with prior infections received 3 vaccine doses, 33% received 2 doses, and 5% received an unknown number of doses. The university did not screen asymptomatic persons with an infection <90 days before because of the likelihood of false positives. 

**Table 1:** Characteristics of population completing isolation in study of students in a university-managed isolation program, January 1–February 11, 2022*

Characteristic	No. (%) persons by days since last negative test				Total no. (%), N = 323
	≤4 d, n = 181	5–9 d, n = 48	≥10 d, n = 93	Unknown, n = 1	
Self-reported symptoms before or at diagnosis					
No	51 (28)	17 (35)	46 (49)	1 (100)	115 (36)
Yes	130 (72)	29 (60)	46 (49)	0 (0)	205 (63)
Unknown	0 (0)	2 (4)	1 (1)	0 (0)	3 (1)
Prior infection >90 d					
No	171 (94)	46 (96)	84 (90)	1 (100)	302 (93)
Yes	10 (6)	2 (4)	9 (10)	0 (0)	21 (7)
No. vaccine doses					
1	3 (2)	0 (0)	6 (6)	0 (0)	9 (3)
2	38 (21)	16 (33)	31 (33)	1 (100)	86 (27)
3	136 (75)	30 (62)	54 (58)	0 (0)	220 (68)
4	1 (1)	1 (2)	0 (0)	0 (0)	2 (1)
Unknown	3 (2)	1 (2)	2 (2)	0 (0)	6 (2)

*Category totals may not add to 100% because of rounding.

We categorized vaccinations into 1–4 doses. In general, a non-mRNA vaccine primary series counted as 1 dose, an mRNA vaccine primary series as 2 doses, and a booster as an additional dose. Two students reported receiving 2 boosters, giving each a total of 4 doses. Only doses administered >14 days before diagnosis were counted toward the total (*18*). The breakdown of doses was as follows: 3% of persons had 1 dose, 27% had 2 doses, 68% had 3 doses, and 1% had 4 doses; 2% had missing data (Appendix Table). RAT positivity duration, and thus isolation time if requiring a negative exit RAT to leave isolation, is dependent on where a person is in their infection course when COVID is diagnosed. To address this consideration, we used the time since the last negative test as an approximation of the time since infection; 56% of persons tested negative <4 days before diagnosis, 15% 5–9 days before, and 29% >10 days before. One person had missing data. The ≤4 days group represents students compliant with university twice-weekly screening policy, the 5–9 day group a mix of noncompliant routine screeners and arrival screeners, and the >10 day group arrival screeners.

To calculate the percent still positive on day 5 and beyond, we dropped 1 person with an unknown last negative test time and 7 persons who initially tested inconclusive but used the subsequent positive test date as the isolation start; the final dataset comprised 315 persons. Among twice-weekly screeners, 47% of all diagnosed (n = 177) remained positive on day 5, 22% on day 6, 8% on day 7, and 1%–2% on days 8–13 (Figure, panel A). Among students last testing negative 5–9 days before diagnosis, 28% of all diagnosed (n = 47) remained positive on day 5, 17% on day 6, 6% on day 7, and 2%–4% on days 8–9 (Figure, panel B). Students last testing negative >10 days before diagnosis (n = 91) had similar daily positivity rates to the 5–9 day group’s (Figure, panel C).

**Figure:**
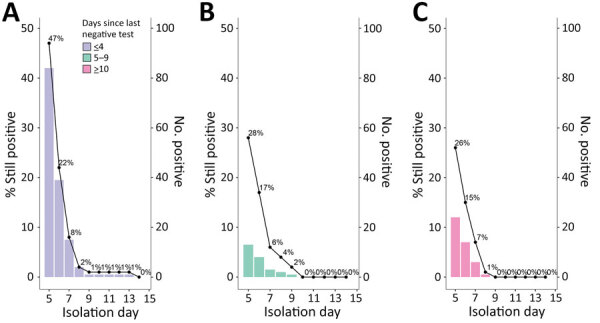
Rapid antigen testing results by isolation day and positivity duration by days since the last negative test category in study of students isolated for positive SARS-CoV-2 results. Left axis shows percent still positive of the original study population; right axis shows the number tested positive on each isolation day. A) Last negative test ≤4 days earlier (N = 177). B) Last negative test 5–9 days earlier (N = 47). C) Last negative test ≥10 days before the earliest test (inconclusive or positive) (N = 91). One person was removed due to missing last negative test data, and 10 persons were removed due to testing inconclusive initially but counted the first positive test as day 0.

To evaluate possible prognostic variables for RAT positivity duration, we conducted a survival analysis using an AFT lognormal regression model. We subset the final dataset to exclude those with 1 (n = 8), 4 (n = 2), or an unknown number (n = 6) of vaccine doses because of small category sizes, a missing PCR cycle threshold (Ct) value at diagnosis because of an external PCR test or home RAT (n = 27), a missing symptom status (n = 2), and receipt of an international vaccine (n = 8), resulting in a final sample of 263 persons. We included time since the last negative test category as a covariate to account for possible confounding, because persons in different infection stages would necessarily experience different RAT positivity durations. We also included symptom status, PCR Ct value, and prior infection >90 days before symptom onset as covariates. We created a new variable combining the number of vaccine doses (2 or 3) and the time since the last dose (<5 months or >5 months) (*19*). All students who had received 3 vaccine doses received their last dose <5 months except for 1 student. Finally, we included the primary series vaccine brand grouped into mRNA vaccines (Pfizer-BioNTech, https://www.pfizer.com, and Moderna, https://www.modernatx.com) and J&J/Janssen (https://www.jandj.com). We determined regression results (Table 2) and RAT positivity duration distribution for each covariate category (Appendix Figure 1) excluding time since last negative test (Figure). We found that having a last negative test >10 days prior was significantly associated with a 15% shorter RAT positivity duration (ETR 0.85, 95% CI 0.75–0.96) compared with having a last negative test <4 days prior. Being symptomatic was significantly associated with a 13% longer RAT positivity duration (ETR 1.13, 95% CI 1.02–1.25). Having a prior infection >90 days was significantly associated with a 50% shorter RAT positivity duration (ETR 0.50, 95% CI 0.33–0.76). Receipt of 3 vaccine doses was significantly associated with a 20% longer RAT positivity duration (ETR 1.20, 95% CI 1.04–1.39) compared to the 2 doses >5 months group. The results for other covariates were not significant.

**Table 2:** Event time ratios of the association between covariates in study of students in a university-managed isolation program, January 1–February 11, 2022*

Covariate	Sample size	ETR (95% CI)	p value
Time since last negative test, d			
<4†	155	NA	NA
5–9	40	0.88 (0.77NA1.01)	0.065
>10	68	0.85 (0.75NA0.96)	0.008
Symptoms at diagnosis			
N	104	NA	Referent
Y	159	1.13 (1.02NA1.25)	0.016
Ct value at diagnosis	263	1 (0.99NA1)	0.378
Prior infection >90 d			
N†	244	NA	NA
Y	19	0.5 (0.33NA0.76)	0.001
No. dose/time since last dose			
2 doses / >5 mo†	44	NA	NA
2 doses / <5 mo	31	1.29 (0.97NA1.73)	0.083
3 doses	188	1.2 (1.04NA1.39)	0.012
Primary vaccine brand			
Janssen/Johnson & Johnson†	24	NA	NA
mRNA	239	1.21 (0.89NA1.65)	0.219

*N = 263 persons who were fully vaccinated with Pfizer-BioNTech (https://www.pfizer.com), Moderna (https://modernatx.com), or Janssen/Johnson & Johnson (https://jnj.com), did not additionally receive an international vaccine, and did not have a missing Ct value or symptom status. ETR >1 is associated with prolonged RAT positivity duration compared to the reference group. An ETR <1 is associated with a decreased RAT positivity duration. Ct, cycle threshold; Dx, diagnosis; ETR, event time ratio; mRNA, either of the mRNA vaccines from Pfizer or Moderna; NA, not applicable; RAT, rapid antigen test.

## Discussion

We analyzed data from a mandatory daily RAT program among university students in isolation to assess the percent still positive on day 5 and beyond and determine possible prognostic factors for RAT positivity duration. In addition, we approximately accounted for time since infection by stratifying our analysis by the time since last negative test. We found a day 5 positivity of 47% in the twice-weekly screening group and 26%–28% in the less frequently screened groups (Figure, panels A–C). For all groups, positivity approximately halved with each additional day. Those results align with the expectation that more frequent screeners received their diagnosis earlier in their infection, thus experiencing a longer isolation. Our findings are similar to results reported in other analyses of managed isolation programs, although most did not conduct daily mandatory testing (*3*; J.A. Hay et al., unpub. data; E. Landon et al., unpub. data; S.B. Nelson et al., unpub. data). Those studies reported RAT positivity of 31%–58% on days 5–9 of isolation and PCR positivity of 39%–52% on day 5, 25%–33% on day 6, and 13%–22% on day 7 .

Two recent cohort studies comparing RAT and culture positivity found a 100% negative predictive value and 50% positive predictive value 4–6 days (n = 14) (*20*) and 6 days (n = 17) (L.A. Cosimi et al., unpub. data, https://doi.org/10.1101/2022.03.03.22271766) after diagnosis. Day 6 culture positivity was 11%–35% depending on the isolation start definition. A separate study found that 25% of persons still had culturable virus at day 8 (J. Boucau, unpub.data, https://doi.org/10.1101/2022.03.01.22271582). The combined results suggest that a negative exit RAT toward the end of isolation is strongly indicative of culture negativity, whereas a positive exit RAT is only sometimes associated with culture positivity (and likely infectiousness). Thus, managed isolation programs face the choice of whether and how to integrate RAT exit testing. In our study, negative RAT tests on day 5 enabled 78%–85% of students to confidently leave isolation 1 day early and negative RAT tests on day 6 to leave on time. For the 15%–22% who remained RAT positive on day 6, some unknown percentage likely remained infectious; the percentage remaining positive dropped to 6%–8% on day 7. We defined the isolation start as the initial test date; however, CDC guidelines define it as the initial test or the symptom onset. Persons using symptom onset as their isolation start may have longer RAT positivity durations than those we measured in our study, strengthening the argument for the use of exit tests, particularly given the innate subjectivity of self-reported symptoms. In addition, we note that the young age of our study population may have meant faster viral clearance than for the general population. An appropriate balance, particularly in the case of high-density settings such as university dormitories in which outbreaks can quickly spread, may be to use exit testing beginning on day 5 to end isolation and, for those still testing positive, remain in isolation until day 7 and continue masking until day 10.

A negative test >10 days before diagnosis, symptom status, prior infection >90 days before diagnosis, and receipt of 3 vaccine doses were significantly associated with RAT positivity duration in our survival analysis (Table 2). Results for the other covariates were not significant. For the last negative test covariate, we observed an association with shorter duration time for the >10 days and 5–9 days groups compared with the <4 days group, although only the difference in ETR for the >10 days group was significant. The relationship between less frequent screening and shorter RAT positivity duration is intuitive; those persons are more likely to receive a diagnosis later in the infection. Reporting symptoms before or at diagnosis was significantly associated with longer RAT positivity duration. Symptomatic persons may receive diagnosis earlier in their infection, even when participating in routine screening, resulting in longer RAT positivity. Experiencing a prior infection >90 days earlier was significantly associated with decreased RAT positivity duration. In a highly vaccinated population, having a previous infection may confer greater immunity than not having one (*21*), reducing the RAT positivity duration.

Receipt of 3 vaccine doses was significantly associated with a longer RAT positivity duration compared with receipt of 2 doses >5 months before diagnosis, an unexpected finding. This finding was consistent under various formulations of the model during our exploratory phase and could be caused by immunologic or data factors. Another study found that vaccine-boosted persons were twice as likely to test positive on an initial RAT on days 5–10 than unboosted persons, although not all persons tested daily (E. Landon et al., unpub. data). That study suggested boosted persons might develop symptoms earlier due to a faster immune response, leading to speedier detection and longer RAT positivity durations. Accounting for the time since the last negative test in our model would likely reduce some of the bias toward earlier detection of symptomatic persons; however, this explanation remains possible. In addition, the quantity and quality of anti-spike antibody levels substantially differ in 2-dose mRNA recipients shortly after they receive a booster dose, enhancing viral neutralization capacity (*22*). Timely onset of improved humoral and cellular immunity in boosted persons is expected to result in rapid control of the acute infection. After such containment, an apparent delay of viral clearance might result from remaining, potentially antibody-coated, viral particles or infected cells that are gradually cleared. In our study population, 68% of persons were boosted with a third dose >14 days before their positive test (Table 1), occurring on average 50 (IQR 35–61) days earlier. Conversely, it is also possible that selection bias exists among boosted persons in our dataset. Boosted persons who experience breakthrough infections may not mount as strong an immunologic response to the vaccine compared as boosted and exposed persons who do not experience breakthrough infections, leading to relatively longer infection durations. In addition, more persons in the 2-dose groups may have been infected with the Delta variant compared with the 3-dose group. We observed a higher proportion of persons belonging to the 2-dose groups earlier in our study, when Delta still circulated at low levels (Appendix Figure 2). If the incubation period or infection duration differ between Delta and Omicron infections, this could contribute to our findings. Although we did not have access to viral sequence data for our study population, Omicron reached 97% frequency among sequenced samples in New Haven County, Connecticut, by January 1, 2022; the remaining 3% were Delta (*23*). We observed a substantially larger sample size for the 3-dose group (n = 188) than the 2-dose >5 months (n = 44) and <5 months (n = 31) groups. The larger sample may have captured more RAT positivity duration outliers. Finally, our analysis assesses the relationship between these factors and the duration of RAT positivity, not infection. Other unaccounted factors may be associated with both the 3-dose group and RAT positivity duration.

Symptom status only captures self-reported symptoms before or at diagnosis and may not always be related to the subsequent SARS-CoV-2 diagnosis. Three persons reported a symptom onset >10 days before diagnosis. Some asymptomatic persons may have later become symptomatic. Prior infections >90 days earlier included confirmed infections reported in the medical records; prior infections that occurred during breaks or before routine screening began at the university in fall 2021 were likely missed. The PCR Ct value was measured only at diagnosis; some Ct values were missing because participants took external tests or home RATs. Our study population, primarily students 18–22 years of age, may not be representative of the general population because of their youth and likely lower rate of comorbidities. However, it is unlikely that older age groups or those with higher comorbidity rates would experience shorter RAT positivity durations. In addition, daily RAT positivity may change in this population as more time passes since their last vaccine dose. We do not have a full medical history for our study population, and it is possible that some persons may experience longer isolations because of their medical conditions. There could be changes in staff accuracy over time in reading RAT results, which are qualitative in nature, although their training procedures render this less likely. We do not have RAT data for days 1–4 and accounted for this interval-censoring in our analysis. RATs have a lower sensitivity than PCR, reducing the risk that a noninfectious person would remain in isolation but increasing the risk for a false negative (*4*,*5*). RAT positivity, although associated with culturable virus, does not mean that a person is necessarily infectious (*5*–*7*).

Incorporation of exit rapid antigen testing into its managed isolation program enabled the university to tailor isolation durations on the basis of onward transmission risk. When using the positive test collection date as the start of isolation, the university released 53%–74% of students testing negative via RAT 1 day early on isolation day 5, while identifying the 15%–22% of students who remained positive on isolation day 6. Using an earlier symptom onset date as an alternative isolation start would result in higher positivity. The recommended full 5-day isolation period may be too short, especially for persons using symptom onset as their isolation start or those with diagnoses early in their infections. Future research analyzing what, if any, onward transmission has resulted from the recommended 5-day isolation period would further refine our understanding of its suitability. In addition, the risk posed by a still-infectious person released from isolation after 5 days must also be considered in the broader context. In periods of high community incidence, the contribution of still-infectious released persons to onward transmission may be relatively small compared with that of other persons early in their infections. Conversely, in periods of low community incidence, their contribution may be relatively greater. These considerations illustrate the complexity of recommending isolation periods for the general population, but our study adds to evidence that the recommended 5-day isolation period may be too short. Finally, our study highlights the utility of using exit RATs to tailor isolation periods on the basis of risk, especially in dense settings or ones with vulnerable populations.

Appendix Additional information from study of daily rapid antigen testing to tailor university COVID-19 isolation policy.
