# Supplementary material for: Daily Rapid Antigen Exit Testing to Tailor University COVID-19 Isolation Policy
Source: Emerg Infect Dis. 2022 Dec;28(12):2455–62. doi: 10.3201/eid2812.220969 (PMC9707582; doi:10.3201/eid2812.220969)
Supplement: Appendix — Additional information from study of daily rapid antigen testing to tailor university COVID-19 isolation policy. [file 22-0969-Techapp-s1.pdf]

Article DOI: https://doi.org/10.3201/eid2812.220969

Daily Rapid Antigen Exit Testing to Tailor University COVID-19 Isolation Policy

Appendix

Appendix Table. Vaccination history of the population of a study of rapid antigen testing for tailoring university COVID-19 isolation policy*

Vaccine brands				Given ≥ 14 days before diagnosis?				
Prior primary series	Primary series	Booster	Second booster	Primary series	Booster	Second booster	No. doses	No. persons
NA	Pfizer	Pfizer	NA	Yes	Yes	NA	3	131
NA	Moderna	Moderna	NA	Yes	Yes	NA	3	44
NA	Pfizer	Moderna	NA	Yes	Yes	NA	3	35
NA	Pfizer	NA	NA	Yes	NA	NA	2	26
NA	Pfizer	Pfizer	NA	Yes	No	NA	2	19
NA	JJ	Moderna	NA	Yes	Yes	NA	2	14
NA	JJ	Pfizer	NA	Yes	Yes	NA	2	8
Sinopharm	Pfizer	NA	NA	Yes	NA	NA	3	5
NA	Moderna	Moderna	NA	Yes	No	NA	2	5
NA	Moderna	NA	NA	Yes	NA	NA	2	5
NA	JJ	JJ	NA	Yes	Yes	NA	2	3
NA	Moderna	Pfizer	NA	Yes	Yes	NA	3	3
NA	JJ	Unknown	NA	Yes	Unknown	NA	Unknown	2
NA	Pfizer	Moderna	NA	Yes	No	NA	2	2
NA	Pfizer	Unknown	NA	Yes	Unknown	NA	Unknown	2
NA	Sinopharm	NA	NA	Yes	NA	NA	1	2
NA	Sinovac	NA	NA	Yes	NA	NA	1	2
Sinovac	Pfizer	NA	NA	No	NA	NA	1	1
Unknown	Pfizer	NA	NA	Yes	NA	NA	3	1
NA	AZ	Pfizer	NA	Yes	No	NA	1	1
NA	JJ	Moderna	NA	Yes	No	NA	1	1
NA	JJ	Pfizer	NA	Yes	No	NA	1	1
NA	JJ	NA	NA	Yes	NA	NA	1	1

Vaccine brands				Given $\geq$ 14 days before diagnosis?				
Prior primary series	Primary series	Booster	Second booster	Primary series	Booster	Second booster	No. doses	No. persons
NA	Moderna	Pfizer	NA	Yes	No	NA	2	1
NA	Moderna	Unknown	NA	Yes	Unknown	NA	Unknown	1
NA	Pfizer	Moderna	Pfizer	Yes	Yes	Yes	4	1
NA	Pfizer	Pfizer	Pfizer	Yes	Yes	Yes	4	1
NA	Pfizer	Pfizer	Unknown	Yes	Yes	Unknown	Unknown	1
NA	Pfizer	Unknown	NA	Yes	No	NA	2	1
NA	Pfizer	Unknown	NA	Yes	Yes	NA	3	1
NA	Sinopharm	Pfizer	NA	Yes	Yes	NA	2	1
NA	Sinovac	Moderna	NA	Yes	Yes	NA	2	1

*JJ, Janssen; NA, not applicable.

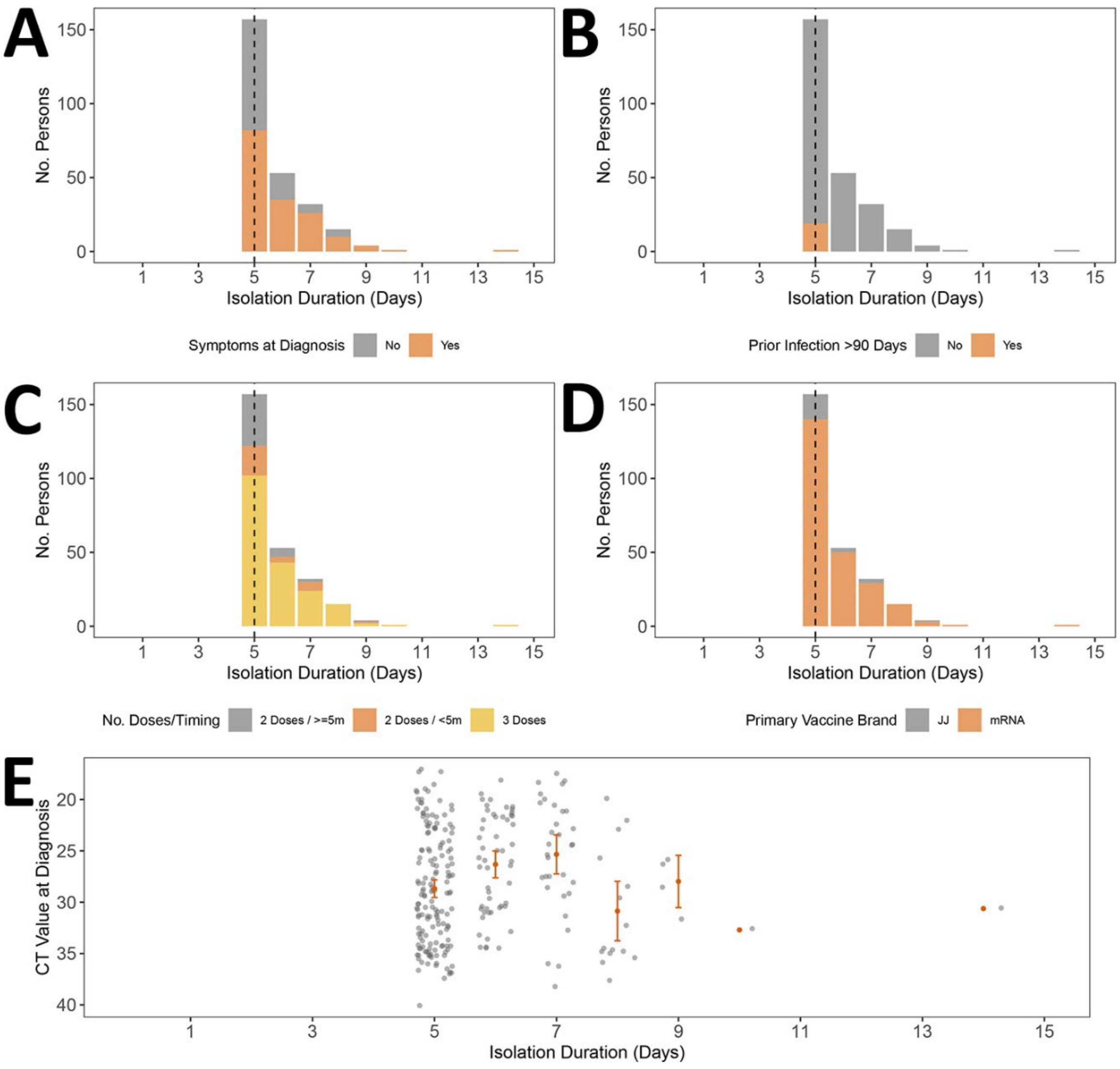

Appendix Figure 1. Relationship between model covariates and RAT positivity duration. We included 263 persons in the survival model analysis (Table 2). RAT positivity duration is measured as the number of days from testing positive or inconclusive to testing negative. The dashed vertical line indicates day 5, the first day of rapid antigen testing. A) RAT positivity duration by symptom status. (B) Positivity duration relative to whether prior infection was in the past 90 days or earlier. C) Positivity duration relative to the number of vaccine doses and time since the last dose. (D) Positivity duration by primary vaccine brand. (E) Positivity duration by Ct value at diagnosis**.** The inverted y-axis shows that lower Ct values correspond to higher viral loads. Orange dot indicates mean and bars indicate 95% CI. Ct, cycle threshold; RAT, rapid antigen test.

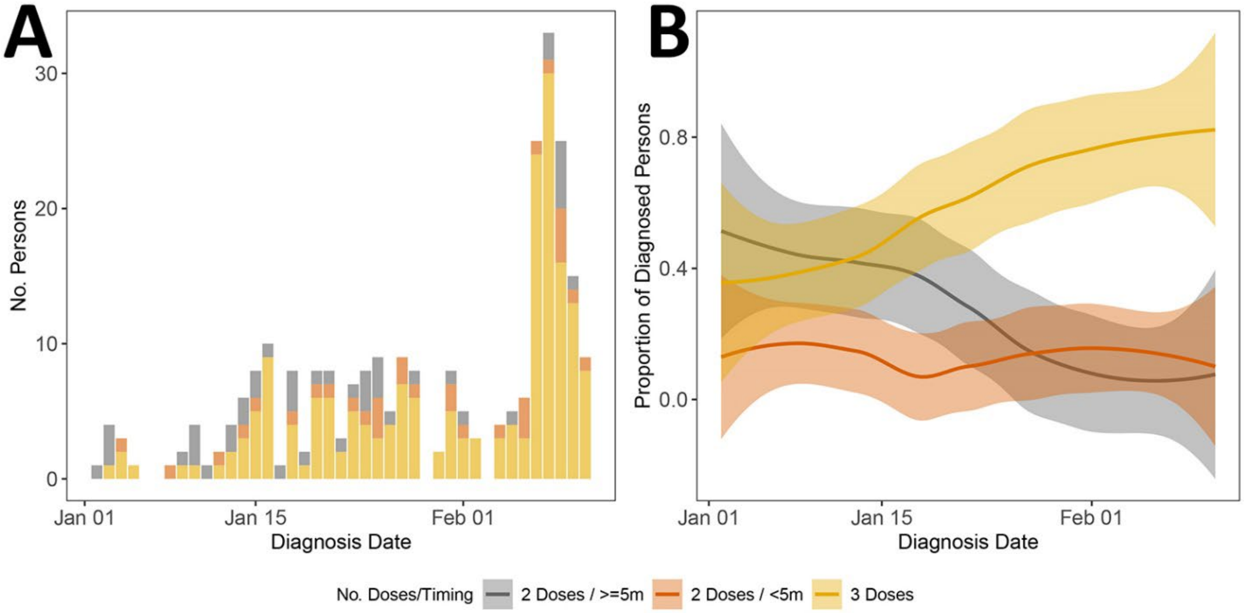

Appendix Figure 2. Number of vaccine doses and time since the last dose by diagnosis date. We included 263 persons in the survival model analysis (Table 2). The diagnosis date is the date of the first positive or inconclusive test. A) Number of persons in each number and timing of vaccine dose category over time by diagnosis date. B) Smoothed proportion with 95% CI of total daily diagnosed persons in (A) belong to each number and timing of vaccine dose category over time.
